# Ultrafast Synthesis of Ni-MOF in One Minute by Ball Milling

**DOI:** 10.3390/nano8121067

**Published:** 2018-12-18

**Authors:** Ren Zhang, Cheng-An Tao, Rui Chen, Lifang Wu, Xiaoxuan Zou, Jianfang Wang

**Affiliations:** 1 College of Chemistry, Key Laboratory of Environmental Friendly Chemistry and Application in Ministry of Education, Xiangtan University, Xiangtan 411105, China; 13574092586@163.com (R.Z.); wulifangyou@163.com (L.W.); 2 College of Liberal Arts and Science, National University of Defense Technology, Changsha 410073, China; Chenrui13@nudt.edu.cn

**Keywords:** metal-organic frameworks, ball milling, solvent-free, mechanochemistry, liquid assisted grinding

## Abstract

A mechanical ball milling method for ultrafast synthesis of a nickel-based metal organic framework (Ni-MOF) has been proposed. The Ni-MOF was successfully synthesized in merely one minute without any solvent, additives, or preliminary preparation. The effect of milling time, mechano-frequency, type of assistant liquid, and amount of assistant water were systematically explored. It was found that the product can be obtained even only at a mechano-frequency of 10 Hz within one minute without any external solvent-assist, which indicated that the crystal water present in the nickel precursor was sufficient to promote MOF formation. Increasing the milling time, raising the mechano-frequency, and the addition of assistant solvent could promote the reaction and increase the yield. The method is rapid, highly efficient, eco-friendly, and has great scalability. The product generated within merely one minute even exhibited high capacitance.

## 1. Introduction

Metal-organic frameworks (MOFs) [1,2] have attracted intense attention during the past two decades due to their intriguing structural properties, which led to many potential applications, including gas storage [3], separations [4], catalysis [5], and sensing [6,7,8], etc. Recently, MOFs have been proven to be useful for electrochemical energy storage and considered as potential electrode material for supercapacitors because of their very large surface area, adjustable pore size, controllable microporous structure, and special structures with potential pseudo-capacitive redox centers [9,10,11,12].

Among them, nickel-based MOFs have get more interest not only because they have been demonstrated to have great application prospects for supercapacitors, but also because they could be the template/precursor for unique metal oxide and carbon materials with specific structure, especially the Ni_3_(BTC)_2_·12H_2_O (BTC = 1,3,5-benzenrtricarboxylic acid). Kong’s group [13] demonstrated that the hydrothermally-synthesized Ni_3_(BTC)_2_·12H_2_O had high specific capacitance of 726 F/g. Wang et al. [14] took the Ni-MOF as a precursor to prepare mesoporous metal oxide by calcining the precursor in the air, and the prepared nickel oxide (NiO) had high-capacitance retention at high scan rate and exhibited excellent cycle-life stability due to its special mesoporous character with bimodal size distribution. Chen et al. [15] synthesized large-scale of multiwalled carbon nanotubes using Ni_3_(BTC)_2_·12H_2_O as precursor. However, the synthesis processes of Ni-MOFs normally require bulk solvents, high temperatures, and long reaction times. For instance, Wang et al. [14] synthesized Ni_3_(BTC)_2_·12H_2_O in a Teflon-lined autoclave and heated at 200 °C for 24 h, Du et al. [16] synthesized Ni-BTC MOF in a Teflon-lined autoclave and heated to 150 °C for 12 h in *N*,*N*-dimethyl formamide (DMF), and Kong’s group [13] prepared Ni-MOF at a lower temperature of 105 °C in DMF but for longer time (up to two days). Although Jin et al. [17] described a simple solution-phase method for the synthesis of Ni_3_(BTC)_2_·12H_2_O under room temperature in a short time, an organic linker must be deprotonated, and an organic solvent was also used. Therefore, a simple, green, rapid, and energy-efficient route to generate Ni-based MOFs without high temperatures or bulk solvents is still highly desirable.

Mechanochemistry, i.e., chemical synthesis enabled or sustained by mechanical force [18], has been recently introduced as an alternative to conventional MOF syntheses [19,20]. Mechanochemical synthesis of MOFs enable avoiding bulk solvents, high temperature, and/or corrosive reagents frequently employed in solution synthesis. Importantly, it is even possible to be more rapid and efficient than solvent-based methods. Since the synthesis of the moderately porous copper(II) isonicotinate MOF in pioneering work reported by James’ group [21], milling procedures have been successfully applied for the synthesis of several popular MOF materials, such as zeolitic azolate frameworks [22,23], rare-earth(III) metal−organic frameworks [24], isoreticular metal-organic frameworks (IRMOFs) [25], iron(III) trimesate MIL-100 (MIL = Materials of Institut Lavoisier) [26], MOF-74 [27], Hong Kong University of Science and Technology (HKUST)-1 [28], Cu_2_I_2_(triphenylphosphine)_2_(L)_n_ (*n* = 1, 2) [29], copper-based MOF-505 [30], and UiO-66 (UiO = University of Oslo) and UiO-67 derivatives [31,32,33]. Previously, Pichon and James [21] described a survey of the potential reactions between Ni(OAc)_2_, Ni(NO_3_)_2_, NiSO_4_, and H_3_BTC under mechanochemical solvent-free conditions and, unfortunately, only grinding nickel sulfate with H_3_BTC gave a partial reaction. Consequently, the ability of mechanochemistry to access Ni-MOFs rapidly, has remained insufficient explored.

**Figure 1:**
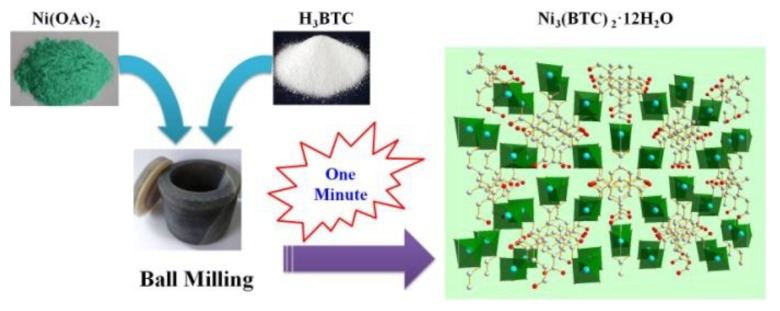
Schematic representation of the ball milling method for the rapid synthesis of Ni_-_MOF.

Herein, we report the ultrafast synthesis of Ni-MOF by a mechanical ball milling method (Figure 1). Notably, Ni-MOF could be obtained in merely one minute without bulk solvent, additives, or any preliminary preparation. The structure and the morphology of Ni-MOF were confirmed by powder X-ray diffraction (PXRD), and scanning electron microscopy (SEM), respectively. Interestingly, the Ni-MOF was formed by fast crystallization within one minute, after which there was no apparent change in the yield and crystallinity, even after 180 min. The effect of milling time, mechano-frequency, kind of assistant liquid, amount of assistant water were systematically explored. The scalability of the ball milling method were also investigated. The generated Ni-MOFs exhibit high capacitance as high as 640 F/g at the current density of 1 A/g. To the best of our knowledge, this is the first example of ultrafast synthesis of Ni-MOF by ball milling.

## 2. Materials and Methods

### 2.1. Materials

Nickel(II) acetate tetrahydrate [Ni(OAc)_2_·4H_2_O, AR] was purchased from Tianjin Fenchuan Technology Co. Ltd. (Tianjin, China) and benzene-1,3,5-tricarboxylic acid (H_3_BTC, 99%) was purchased from J&K Scientific Ltd. (Beijing, China). DMF(AR) was purchased from Tianjin Hengxing Chemical Preparation Co. Ltd. (Tianjin, China), methanol (MeOH, AR) was purchased from Sinopharm Chemical Reagent Co. Ltd. (Beijing, China), and ethanol (EtOH, 99.7%) was purchased from General-Reagent (Shanghai, China). Pure water was homemade.

### 2.2. Synthesis of Ni-MOF

Ni-MOFs were synthesized by ball milling of of 2:3 (molar ratio) mixtures of H_3_BTC and Ni(OAc)_2_·4H_2_O at varied frequency from 10 Hz to 50 Hz. In a typical reaction, about one gram of the H3BTC and metal precursor mixture was ball milled for various times (1, 5, 30, 60, and 180 min) in a agate vial (80 mL) using a QM-QX0.4L mill (Miqi Instrument Equipment Co., Ltd., Changsha, China) with the addition of specific amount of water (0, 0.5, 1, or 2 mL) or 1 mL of other organic solvent (MeOH, EtOH, and DMF) and increased the feed rate by three times and five times. The ball-to-powder mass ratio was consistently kept at about 5:1 for all experiments. The products were scraped off the jar walls, washed with water and ethanol trice, then the supernatant was removed by centrifugation, and the solid product was dried at 60 °C in an oven for 12 h. The enlarged scale reaction was performed with three or five times of the H_3_BTC and metal precursor mixture. The yield was calculated according to the following equation based on the number of moles of Ni(II).

$$\mathrm{Yield}=\frac{n_{\mathrm{Ni}\left(\mathrm{II}\right)\;\mathrm{in}\;\mathrm{Ni}-\mathrm{btc}}}{n_{\mathrm{Ni}{\left(\mathrm{OAc}\right)}_{2}}}\times 100\%$$

### 2.3. Characterization

FTIR spectra were recorded in the range of 400–4000 cm^−1^ on a PerkinElmer Spectra Two FT-IR spectrophotometer (Waltham, MA, USA) with an attenuated total reflectance (ATR) accessory. The milled samples were analyzed by powder XRD on a Tri III powder diffractometer (Rigaku, Tokyo, Japan) using Cu Kα radiation between 8° and 60° with a scan rate of 5°/min. Thermogravimetric analyses (TGA) were performed on a STA6000 thermal analyzer (PerkinElmer, Waltham, MA, USA) under N_2_ with a heating rate of 4 °C/min. Nitrogen adsorption-desorption isotherms, pore size distributions and surface areas of the samples were measured via N_2_ adsorption-desorption at 77 K on a BEL SORP-mini II surface area and porosity analyzer (Bel Japan Inc., Osaka, Japan). Before measurement, the samples were activated at 60 °C for 12 h. The morphology of the sample was observed by a SEM Model S-4800 (Hitachi, Tokyo, Japan).

All electrochemical measurements were done in a three-electrode experimental setup. A platinum wire electrode and a saturated Ag/AgCl electrode were used as the counter and reference electrodes, respectively. All the electrochemical measurements were carried out in 6 mol/L KOH aqueous electrolyte using a CHI660C (Shanghai Chenhua Apparatus, Shanghai, China) electrochemical workstation.

## 3. Results and Discussion

### 3.1. Synthesis of Ni-MOF

#### 3.1.1. Effect of Grinding Time

**Figure 2:**
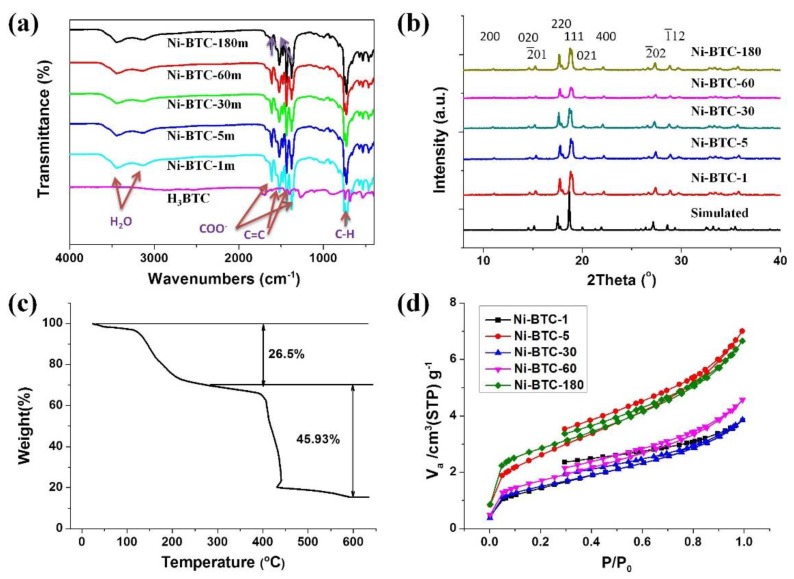
Characterizations of the Ni-BTC samples obtained at various reaction times. (**a**) FTIR spectra, (**b**) XRD patterns, (**c**) TGA curve of Ni-BTC-1m, and (**d**) Nitrogen adsorption and desorption.

Firstly, the Ni-MOF Ni_3_(BTC)_2_·12H_2_O was synthesized by grinding of 2:3 (molar ratio) mixtures of H_3_BTC and Nickel(II) acetate tetrahydrate [Ni(OAc)_2_·4H_2_O] with water-assist under mechano-grinding at 50 Hz for a specific time (1, 5, 30, 60, and 180 min). The products are denoted as Ni-BTC-1 m, Ni-BTC-5 m, Ni-BTC-30 m, Ni-BTC-60 m, and Ni-BTC-180 m, respectively. There is no apparent change in the yield, which only varied from 66% to 72% (Table S1). The Fourier transform infrared (FTIR) spectra of different Ni-MOFs are very similar. These spectra clearly show the vibrational bands of the waters around 3500 and 3200 cm^−1^, which suggests there are crystallization waters in the product (Figure 2a). Additionally, there is no band at around 1710 cm^−1^ (which is the characteristic of protonated carboxylic groups) observed in Ni-MOFs curves, suggesting the absence of protonated carboxylic groups in the product.

The XRD pattern shows the characteristic diffraction peaks of Ni_3_(BTC)_2_·12H_2_O (Figure 2b), which matches well with the result of the reported literature [14,16,17,34], and the simulated pattern for [Ni_3_(BTC)_2_·12H_2_O] was based on single-crystal data from the Cambridge Crystallographic Data Centre (CCDC) [35]. The generated product in only one minute has already shown good crystallinity. To evaluate the effect of grinding time on the size of Ni-MOF crystals, the full width at half maxima of peaks (FWHM) at 17.7° corresponding to the (220) lattice plane were listed in Table S2. The average size of Ni-MOF crystals was inversely proportional to the FWHM. The FWHM decreases slightly, at first, and then increases, and finally decreases along with grinding time, indicating that the average size of Ni-MOF crystals changed slightly along with the grinding time.

To further confirm the composition of the Ni-MOF, the thermogravimetric analyses (TGA) were performed under air with a heating rate of 4 °C/min. There are two different stages of weight loss in the TGA curve of Ni-MOF-1m, as shown in Figure 2c. In the first stage, weight loss of 27.35 wt % from 100 °C to 250 °C could be ascribed to the loss of crystallization water molecules. The second sharp weight loss started from 250 °C and ended at 400 °C, due to the decomposition of the organic frameworks, and the final residue was NiO [17]. These evidences further prove that the coordinated or adsorbed H_2_O molecules existence in the Ni-MOFs. This result is in conformity to the FTIR data. Additionally, the weight loss is in agreement with the chemical formula Ni_3_(C_6_H_3_(COO)_3_)_2_·12H_2_O.

In addition, nitrogen adsorption-desorption isotherms, the specific surface area and pore structures of Ni-MOFs were studied by surface area and porosity analyzer, and the results are shown in Figure 2d and Table S3. The Brunauer–Emmett–Teller (BET) specific surface area of Ni-MOF-1m is only 4.85 m^2^/g, after the longer reaction time, the specific surface area of Ni-MOF-180m reaches the maximum of 10.08 m^2^/g. The average pore sizes of Ni-MOFs are about 4.6 nm except Ni-MOF-180m (4.1 nm). According to the Barrett–Joyner–Halenda (BJH) analysis of the Ni-BTC samples obtained at various reaction times, as shown in Figure S1, the pore sizes in the micropore range are around 1.7 nm for all MOF samples. However, the pore sizes in the mesopore range are different, and it is inferred that the porosity comes from the interparticle voids, which is different due to the varied morphology of the MOFs.

**Figure 3:**
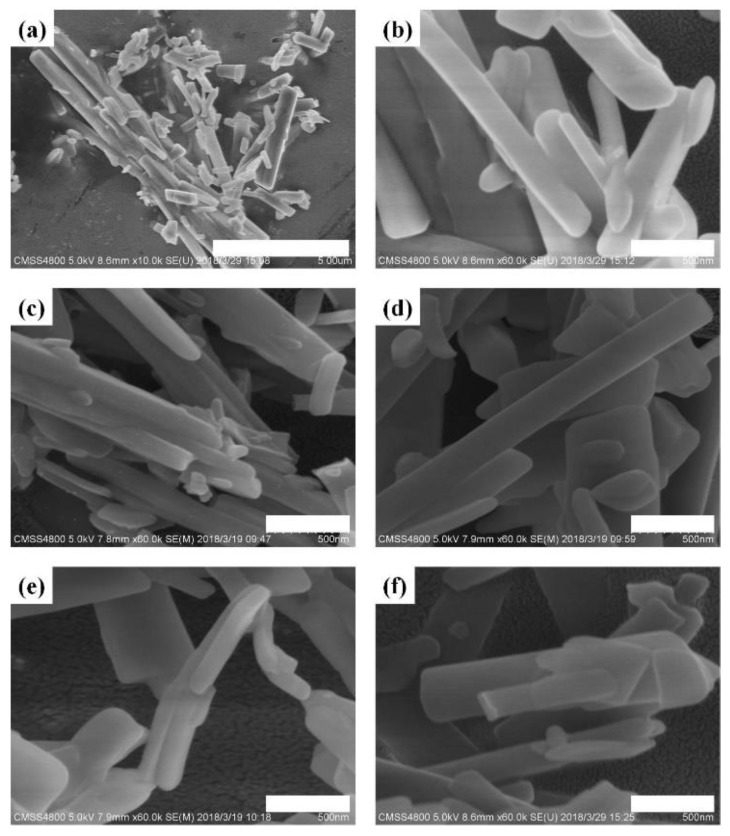
SEM images of the Ni-BTC samples obtained at various reaction times. (**a**,**b**) Ni-BTC-1 m, (**c**) Ni-BTC-5 m, (**d**) Ni-BTC-30m, (**e**) Ni-BTC-60 m, and (**f**) Ni-BTC-180 m. The bar in (**a**) represents 5 μm, and the bars in (**b**–**f**) to represent 500 nm.

Morphological investigations of Ni-MOFs were carried out using a field-emission SEM, and the SEM images are shown in Figure 3. Ni-MOF-1 m exhibits rod-like shapes, but the dispersion of their sizes is very broad. The larger ones have a length and width of 10 μm and 500 nm, respectively, while the smaller ones have a length and width of about 500 nm and 100 nm (Figure 3a,b). The surface of microcrystals of Ni-MOF-1 m looks very smooth. For the products with longer reaction time, there are no apparent change in the morphology (Figure 3c–f and Figure S2), suggesting the success of synthesis of Ni-MOF in merely one minute.

#### 3.1.2. Effect of Mechano-Frequency of Grinding

**Figure 4:**
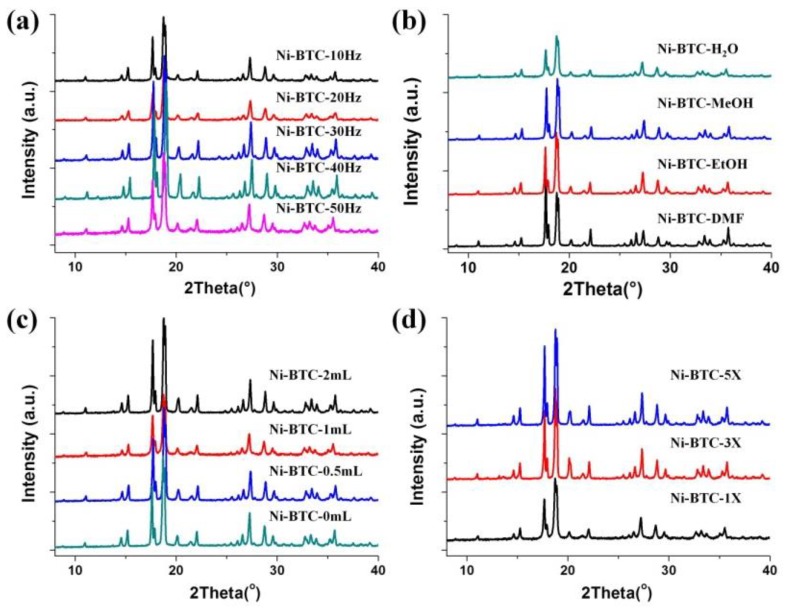
XRD patterns of the Ni-BTC samples obtained at various grinding frequencies (**a**), with addition of different solvents (**b**), with addition of varied amount of water (**c**), and at enlarged scales (**d**).

In addition to the grinding time, the mechano-frequency of grinding is also one key factor of ball-milling conditions. With the assist of water, the products were obtained under different frequencies from 10 Hz to 50 Hz by keeping the reaction time of only for one minute, and they denoted as Ni-BTC-10 Hz, Ni-BTC-20 Hz, Ni-BTC-30 Hz, Ni-BTC-40 Hz, and Ni-BTC-50 Hz, respectively. Their XRD patterns were shown in Figure 4a. All these patterns show the characteristic diffraction peaks of Ni_3_(BTC)_2_·12H_2_O, even the product of Ni-BTC-10Hz. The crystallinity of the products obtained at 10 Hz and 20 Hz are also significantly lower than that obtained at higher frequencies. The yield of Ni-BTC-10Hz is only about 39% (Table S4). When the mechano-frequency increases to 20 Hz, the yield increases to about 60%. To further increase the milling frequency, the yield maintains at about 60–70%.

#### 3.1.3. Effect of Auxiliary Liquid

**Figure 5:**
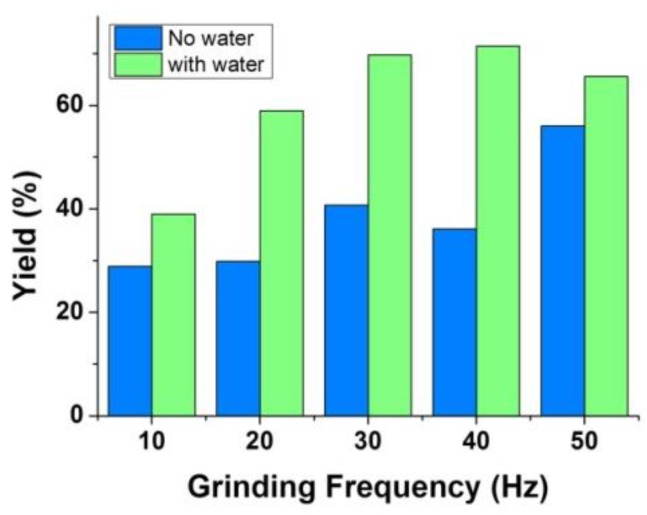
Comparison of yields of Ni-BTC samples obtained with and without addition of water (1 mL).

The effect of the type of auxiliary liquid on the ball milling reaction was studied. In addition to water, the polar solvents MeOH, EtOH, and DMF, commonly used in MOF synthesis, were also explored. The XRD patterns of products were shown in Figure 4b. All of them have similar patterns which match well with the simulated pattern for Ni_3_(BTC)_2_·12H_2_O, suggesting the success of preparation of product in one minute whatever the kind of auxiliary liquid. The yields of the products are between 60% to 70% (Table S5). Water is the best choice among them, considering its environment friendliness. Moreover, the effect of the quantity of water was also investigated. Unexpectedly, it is found that the product can be obtained without water, as shown in Figure 4c, and the yield can achieve about 56% (Table S6). Under the water-assist condition, the yields increase a little to around 65%. This result implies the addition of water is not necessary. Based on this result, the effects of mechano-frequency were explored again under no liquid-assist condition. In the absence of additives, even at a minimum of 10 Hz, we can still obtain a product with good crystallization (Figure S3). However, the yield drops to only about 29% (Table S7). As the frequency increases, the yield increases slightly. Even if the frequency rises to 40 Hz, the yield is only about 40%. When the frequency is raised to 50 Hz (the maximum limit frequency of the instrument), the yield rises to 56%. In contrast to the case with liquid assist (Figure 5), it can be seen that the yield of product with liquid assistance is significantly higher than that without liquid assistance. Under the liquid assist condition, the maximum yield can be achieved at when the frequency is over 30 Hz, while without liquid assistance there is still a large increase in yield at 50 Hz.

#### 3.1.4. Scalability

In addition, we investigated the scalability and expandability of the method. We increased the feed rate by three times and five times, and found that the crystallinity of the product was basically the same (Figure 4d), and the yield was slightly improved (Table S8). This method can be used for bulk preparation of MOFs easily at the gram scale (2.77 g in a run). In general, the method is rapid, high-efficiency, eco-friendly, low cost, and has great scalability.

Recently, a few reports [17,36,37] have also demonstrated the rapid synthesis of MOFs (Table S9). Duan et al. [36] demonstrated the synthesis of hierarchical porous ZIF-8 materials within one min at room temperature by using organic amines as a supramolecular template (organic amine-template), but the bulk organic solvent (methanol) and additives (organic amines) were still required. Jin et al. [17] synthesized Ni_3_(BTC)_2_·12H_2_O by a solution-phase method under room temperature in short time, but the organic linker must to be deprotonated, and the organic solvent were also used. Huang et al. [37] reported the synthesis of F_4_-UiO-66 in 100 s using water-assisted grinding, however, the metal source must be the pre-prepared zirconium clusters. Despite these developments, it should be noted that the work described here represents the first example of ultrafast synthesis of Ni-MOF by ball milling without solvent or any preliminary preparation.

This fast reaction speed is due to the sufficient energy provided by the mechanical force during ball milling. From a thermodynamic point of view, generally, the chemical potential of a substance in the solid state is higher than that of the same one in the liquid state [30,38,39,40]. Thus, the driving force of the formation of Ni-MOF is higher than that of solution-based synthesis method [30]. Therefore, the mechanochemical construction of Ni-MOF can be reacted within a short time. On the other hand, the additional solvents (water, MeOH, EtOH, or DMF) had a good dissolution of the raw materials and, thus, they can boost molecular mobility of the reactants during reaction. Even if no addition of solvent, a small amount of crystal water contained in nickel acetate separates out under the action of ball milling, thereby acting as an auxiliary solvent. Moreover, the acetate ion acts as a base to catalyze the deprotonation of the H_3_BTC, thereby increasing the reaction rate. In the experiment, nickel chloride and nickel nitrate were used as the metal source to carry out the reaction, and no detectable product was found, which proved the inference.

### 3.2. Electrochemical Performance of Ni-MOF

**Figure 6:**
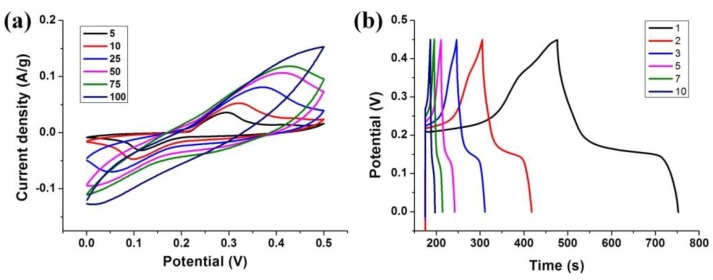
(**a**) Evolution of CVs of Ni-BTC-1 at various scan rates: 5, 10, 25, 50, 75, and 100 mV/s; and (**b**) charge-discharge diagrams of Ni-BTC-1 at different current densities: 1, 2, 3, 5, 7, and 10 A/g.

Finally, to prove that the Ni-MOF produced within one minute has considerable electrochemical performance, cyclic voltammetry (CV) and galvanostatic charge-discharge (GCD) were performed using a three electrode system in 6 mol/L KOH electrolyte and the results were presented in Figure 6. The CV was carried out with a potential range from 0 to 0.5 V at varied scan rates (5, 10, 25, 50, 75, and 100 mV/s). There are a couple of distinct redox peaks could be observed, which are correspond to the reversible redox of the reversible intercalation and deintercalation of OH^-^ ions [13]. These surface faradic redox reactions lead to the typical pseudocapacitive behavior of Ni-MOFs. The GCD curves under different current densities in a potential range of 0–0.45 V are shown in Figure 4b. It can be found that there is a plateau in the potential from 0.2 to 0.25 V at different current densities from 1 to 10 A/g. Such a plateau is ascribed to the redox reaction, indicating the significant contribution of the pseudo-capacitance, which agrees well with the phenomena of CV curves. The specific capacitance C_m_ (F/g) is calculated from the GCD measurement (Figure 4b) according to the following equation [13]:

$$C_{m}=I\Delta t/\Delta E=i\Delta t/\left(m\Delta E\right)$$

where *I* is the discharge current density calculated using *I* = *i*/*m*, *i* is the current and *m* is the active mass of the electrode, Δ*t* is the during time of the discharge curve, and Δ*E* is the potential window of the discharge curve, respectively. The calculated *C*_m_ values of Ni-MOF-1 are present in Figure S4. The specific capacitance can achieve 640 F/g at the current density of 1 A/g, which is comparable to the performance of the hydrothermally-synthesized one [13].

## 4. Conclusions

We have proposed a mechanical ball milling method for ultrafast synthesis of Ni-based metal organic frameworks. The results of XRD show that there is no significant difference in the crystallinity of the products obtained at different reaction times (1–180 min), and the stable product can be obtained even only at macheno-frequency of 10 Hz within one minute without any solvent-assist. Generally, increasing the milling time, raising the mechanical frequency, and the addition of assistant solvent will increase the yield, while the kind of assistant solvent has no evident effect on the yield. The water is the best choice among the solvents, considering the environment friendliness. The method is rapid, highly efficiency, eco-friendly, and has great scalability (at the gram scale in a run).

## Supplementary Materials

The following are available online at http://www.mdpi.com/2079-4991/8/12/1067/s1; Table S1. Yields of Ni-BTC samples obtained at various reaction times; Table S2. FWHM of Ni-BTC samples obtained at various reaction times; Table S3. Surface area and pore structures of Ni-BTC samples obtained at various reaction times; Figure S1. The BJH analysis of the Ni-BTC samples obtained at various reaction times; Figure S2. SEM images of the Ni-BTC samples obtained at various reaction times. (a) Ni-BTC-5 m, (b) Ni-BTC-30 m, (c) Ni-BTC-60 m, and (d) Ni-BTC-180 m; Table S4. Yields of Ni-BTC samples obtained at various grinding frequencies with addition of 1 mL water; Table S5. Yields of Ni-BTC samples obtained with addition of different solvents; Table S6. Yields of Ni-BTC samples obtained with addition of varied amount of water; Figure S3. XRD patterns of the Ni-BTC samples obtained at various grinding frequencies without the water addition; Table S7. Yields of Ni-BTC samples obtained at various grinding frequencies without the addition of water; Table S8. Yields of Ni-BTC samples obtained at enlarged scales; Table S9. Comparison of the synthesis of MOF in previously published reports; Figure S4. Diagram of the specific capacitance of materials at different current densities.
