# Supplementary material for: Ultrafast Synthesis of Ni-MOF in One Minute by Ball Milling
Source: Nanomaterials (Basel). 2018 Dec 18;8(12):1067. doi: 10.3390/nano8121067 (PMC6316361; doi:10.3390/nano8121067)
Supplement: Supplementary file 1 [file nanomaterials-08-01067-s001.pdf]

Supporting Information

Ultrafast Synthesis of Ni-MOF in One Minute by Ball Milling

Table S1 Yields of Ni-BTC samples obtained at various reaction times. .

	Yield(%)
Ni-BTC-1	65.57
Ni-BTC-5	68.31
Ni-BTC-30	67.89
Ni-BTC-60	70.28
Ni-BTC-180	72.68

Table S2 FWHM of Ni-BTC samples obtained at various reaction times. .

	FWHM (°)
Ni-BTC-1	0.173
Ni-BTC-5	0.171
Ni-BTC-30	0.145
Ni-BTC-60	0.163
Ni-BTC-180	0.126

Table S3 Surface area and pore structures of Ni-BTC samples obtained at various reaction times.

	BET surface area ($m^2 g^{-1}$)	Total pore volume ($cm^3 g^{-1}$)	Average pore size (nm)
Ni-BTC-1	4.85	0.0059	4.6
Ni-BTC-5	8.85	0.0108	4.6
Ni-BTC-30	5.28	0.0059	4.5
Ni-BTC-60	6.08	0.007	4.6
Ni-BTC-180	10.08	0.0102	4.1

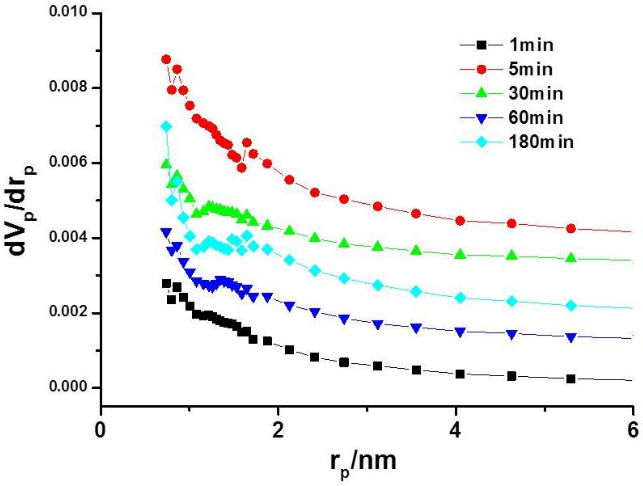

Figure S1. the BJH analysis of the Ni-BTC samples obtained at various reaction times.

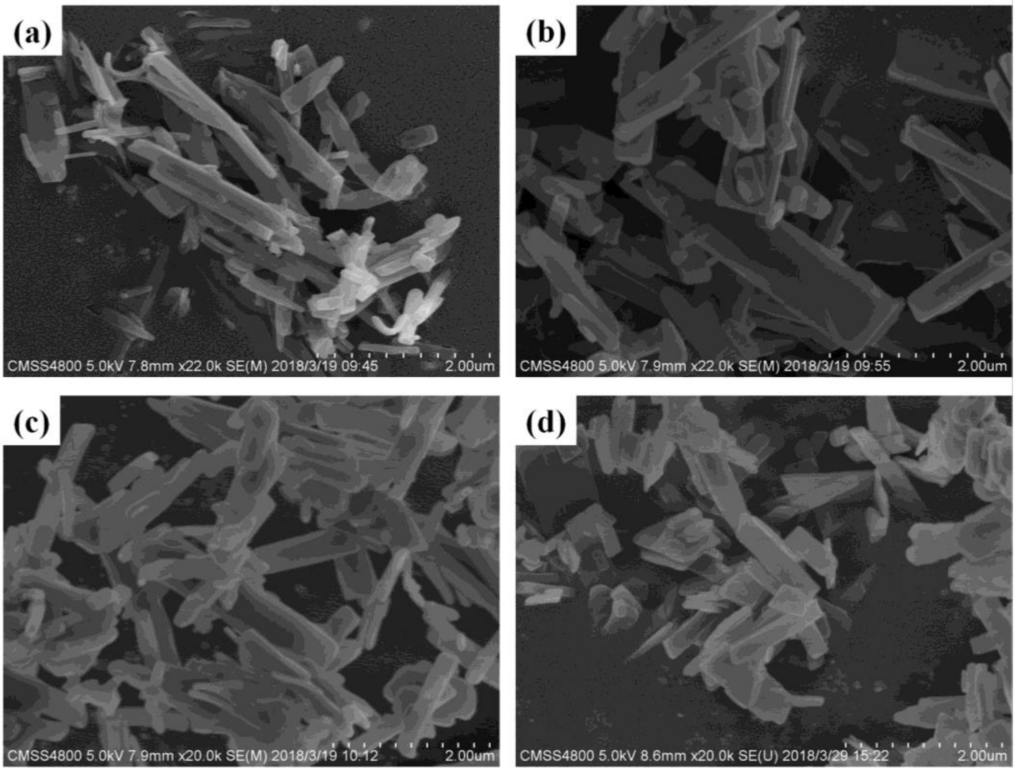

Figure S2. SEM images of the Ni-BTC samples obtained at various reaction times. (a) Ni-BTC-5m, (b) Ni-BTC-30m, (c) Ni-BTC-60m, and (d) Ni-BTC-180m.

Table S4 Yields of Ni-BTC samples obtained at various grinding frequencies with addition of 1mL water. .

	Yield(%)
Ni-BTC-10Hz	38.99
Ni-BTC-20Hz	59.00
Ni-BTC-30Hz	69.75
Ni-BTC-40Hz	71.45
Ni-BTC-50Hz	65.57

Table S5 Yields of Ni-BTC samples obtained with addition of different solvents.

	Yield(%)
Ni-BTC-DMF	67.25
Ni-BTC-EtOH	67.57
Ni-BTC-MeOH	59.69
Ni-BTC-H_2O	65.57

Table S6 Yields of Ni-BTC samples obtained with addition of varied amount of water.

	Yield(%)
Ni-BTC-0mL	56.00
Ni-BTC-0.5mL	66.61
Ni-BTC-1mL	65.57
Ni-BTC-2mL	67.57

Table S7 Yields of Ni-BTC samples obtained at enlarged scales.

	Yield(%)
Ni-BTC-1X	65.57
Ni-BTC-3X	73.43
Ni-BTC-5X	79.76

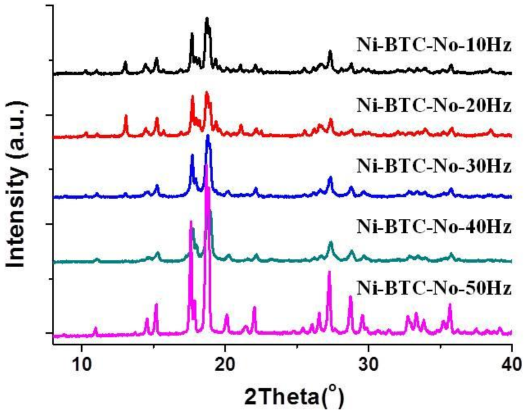

Figure S3. XRD patterns of the Ni-BTC samples obtained at various grinding frequencies without water addition.

Table S8 Yields of Ni-BTC samples obtained at various grinding frequencies without addition of water.

	Yield(%)
Ni-BTC-No-10Hz	28.87
Ni-BTC-No-20Hz	29.89
Ni-BTC-No-30Hz	40.71
Ni-BTC-No-40Hz	36.14
Ni-BTC-No-50Hz	55.99

Table S9. Comparison of the synthesis of MOF in previously published reports.

No.	MOF type	Raw Material	Method	Ref
**1**	$Ni_3(BTC)_2·12H_2O$	$Ni(CH_3COO)_2·4H_2O$, H_3BTC	Ball milling, r.t., 1 min	*This work*
**2**	$Ni_3(BTC)_2·12H_2O$	$Ni(CH_3COO)_2·4H_2O$, H_3BTC, DMF	Hydrothermal, 200°C, 24h	*Electrochimica Acta* **2010**, *55*, 6830-6835.
**3**	$Ni_3(BTC)_2·12H_2O$	$NiCl_2·6H_2O$, Na_3BTC,	Solution-phase method, r.t., 5 min	*Chinese Chemical Letters* **2013**, *24*, 663-667.
**4**	$Ni_3(BTC)_2·12H_2O$	$NiCl_2·6H_2O$, H_3BTC, DMF	Hydrothermal, 105°C, 2day	*Chinese Chemical Letters* **2014**, *25*, 957-961.
**5**	ZIF-8	Zn(NO3)2 , 2-methylimidazole, methanol, diethanolamine	Solution-phase method, r.t., a few minutes	*Science China Materials* **2017**, *60*, 1205-1214.
**6**	F_4-UiO-66	Zicronium(IV)-oxo-hydroxy methacrylate, tetrafluorobenzene-1,4-dicarboxylic acid	Water-assisted grinding, r.t., 100 s	*Chemical Communications* **2017**, *53*, 5818-5821.

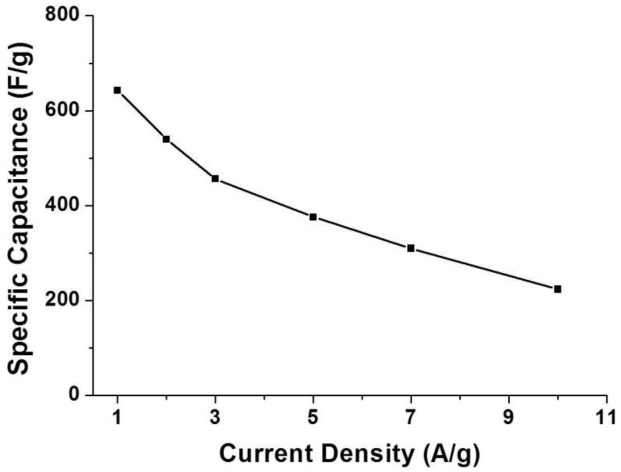

Figure S4. Diagram of the specific capacitance of materials at different current densities.
